# Phytochemical Profile, Antioxidant, Cardioprotective and Nephroprotective Activity of Romanian Chicory Extract

**DOI:** 10.3390/plants10010064

**Published:** 2020-12-30

**Authors:** Alexandra Epure, Alina E. Pârvu, Laurian Vlase, Daniela Benedec, Daniela Hanganu, Ana-Maria Gheldiu, Vlad Al. Toma, Ilioara Oniga

**Affiliations:** 1 Department of Pharmacognosy, Faculty of Pharmacy, “Iuliu Hațieganu” University of Medicine and Pharmacy, 8 V. Babeș Street, 400012 Cluj-Napoca, Romania; ale.epure@gmail.com (A.E.); dbenedec@umfcluj.ro (D.B.); dhanganu@umfcluj.ro (D.H.); ioniga@umfcluj.ro (I.O.); 2 Department of Physiopathology, Faculty of Medicine, “Iuliu Hațieganu” University of Medicine and Pharmacy, 8 V. Babeș Street, 400012 Cluj-Napoca, Romania; 3 Department of Pharmaceutical Technology and Biopharmacy, “Iuliu Hațieganu” University of Medicine and Pharmacy, 8 V. Babeș Street, 400012 Cluj-Napoca, Romania; 4 Department of Pharmaceutical Botany, Faculty of Pharmacy, “Iuliu Hațieganu” University of Medicine and Pharmacy, 8 V. Babeș Street, 400012 Cluj-Napoca, Romania; gheldiu.ana@umfcluj.ro; 5 Department of Chemistry and Chemical Engineering, “Babeș Bolyai” University, 11 Arany János Street, 400028 Cluj-Napoca, Romania; vlad.al.toma@gmail.com

**Keywords:** *Cichorium intybus*, polyphenols, cichoric acid, antioxidant, cardioprotective, nephroprotective

## Abstract

The present study analyzed the methanol extract and tincture obtained from the spontaneous Romanian *Cichorium intybus* species, in order to evaluate polyphenols content and some biological properties. Chromatographic and spectrophotometric methods were used for the analysis of polyphenols and the antioxidant capacity was assessed in vitro with DPPH^●^ (2,2-diphenyl-picrylhydrazil) and FRAP (ferric-reducing antioxidant power) tests. The cardio-protective effects of *Cichorii herba* tincture on myocardial ischemia induced by isoprenaline and nephroprotection on renal failure induced by gentamicin were evaluated on rats. Also, aspartate aminotrasferase (AST), alanine aminotransferase (ALT), creatine kinase-MB (CK-MB) and creatinine clearance (CrCl) were measured. The antioxidant effect was evaluated by determining total oxidative stress (TOS), oxidative stress index (OSI, total antioxidant capacity (TAC), malondyaldehide (MDA), total thiols (SH) and total nitrites and nitrates (NOx). Cichoric acid was the main polyphenolic compound. The extracts had moderate in vitro antioxidant activity but the in vivo antioxidant and anti-inflammatory effects were significant and associated with myocardial and renal dysfunction improvement. The results were attributed to the content of polyphenols in the extracts, for which reason *C. intybus* may be considered an important raw material for pharmaceuticals formulations recommended in the prevention or treatment of heart or kidney diseases.

## 1. Introduction

Medicinal plants offer a wide range of substances that may be sources of therapeutic drugs, characterized by pharmacological activity, efficacy, and safety profile [1].

An important class of natural bioactive compound is represented by polyphenols which are considered secondary metabolites synthesized by plants in their normal development and also as a response to stress factors [2]. As polyphenol compounds, flavonoids and phenolic acids are natural antioxidants that have important roles in protecting biological systems against the harmful consequences of oxidative stress. A valuable source of polyphenolic compounds are the plant species from the Asteraceae family [3]. Among them, *C. intybus*, commonly known as chicory is a perennial herb that spontaneously grows from May to October, and is widespread in all regions of Romania but that can be found in cultivated cultures too [4,5].

The most important compounds identified in *C. intybus* are polyphenols (polyphenolic acids, flavonoids, coumarins, lignans) and terpenoids (sescviterpene lactones, triterpenes). Other compounds present in chicory are saponins, volatile compounds, vitamins, amino acids, fatty acids, carbohydrates (important quantities of inulin occur in roots), organic acids, phytosterols, small amounts of alkaloids and minerals [6].

Polyphenols are predominantly synthesized in the aerial parts and seeds, while the sesquiterpene lactones are found mostly in the roots [7,8].

Between the sesquiterpenes, wich are bitter substances, the guaianolide type are the most common in chicory: lactucin and lactucopicrin and their derivatives [7,9,10].

The phenolic acids from chicory are represented by hydroxycinamic acids that include caffeoylquinic acids and cichoric acid (dicaffeoyltartaric acid) [11,12].

The flavonoids present in cichory consist of flavanols, flavonols, flavones and anthocyanins. The identified compounds are glycosides of quercetin, luteolin, kaempferol, myricetin, apigenin, chrysoeriol, jaquelin, isorhamnetin [13]. The blue color of the perianth is given by anthocyanins: delphinidine and its derivates cyanidin, pelargonidin and petunidin derivatives [14].

In the medical literature, many experimental studies regarding the pharmacological activities of *C. intybus* extracts have been undertaken to asses pharmacological activities such as anti-inflammatory, antimicrobial [13,15,16], antihelminitc [13], antimalarial [17], liver protective [18,19,20], antidiabetic [21], anti-tumor [22], anti-allergic [23] gastro-protective, pancreatic protective, anti-allergic, sedative and diuretic properties etc. [4,13,24]. Various studies highlighted the anti-inflammatory potential of the extracts and described some possible mechanisms of action. For example, compounds from ethyl acetate chicory roots extract were found to inhibit in vitro tumor necrosis factor-alpha (TNF-α) that is linked to cyclooxygenase induction and suppresses the production of pro-inflammatory factor prostaglandin E2 in human colon carcinoma cells [25]. Low mass molecular compounds from methanol extract of *C. intybus* roots showed a significant anti-inflammatory effect against gingival inflammation at a cellular level by inhibition of pathogen bacteria development [26]. In vivo anti-inflammatory effects of *C. radix* ethanol extract were tested successfully on a carrageen-induced rat paw edema model showing a possible mechanism based upon the inhibition of cytokines [13]. Only a few clinical studies have been reported that include *C. intybus* extracts. Chicory was established as a hepatoprotective (whole plant extract, as an ingredient of a combined herbal-based drug, namely Liv-52), by reducing serum levels of alanine aminotransferase (ALT) and aspartate aminotrasferase (AST) and so the ascites and Child–Pugh scores [27]. The positive results of a pilot study showed that *C. intybus* roots extract has a potential role in the management of osteoarthritis [28]. Within *C. intybus* composition, cichoric acid stands out, being one of the polyphenol acids with highest concentration, especially in aerial parts [8]. Cichoric acid represents a promising polyphenol that offers unexplored potential in various disorders because of its properties, such as, antimicrobial anti-inflammatory, lowering blood glucose, vasorelaxant, anti-tumor, anti-obesity etc. [29].

Some researches on species of the Asteraceae family (*Achillea millefolium* L., *Cynara scolymus* L., *Silybum marianum* L., *Tagetes erecta* L. etc.) demonstrated potential cardioprotective activity of the extracts, based on the antioxidant properties of high polyphenols content [30].

The antioxidant properties of polyphenols were also involved in the nephroprotective activity of some vegetable extracts from the *Asteraceae* family, such as *Cynara scolimus* L., *Matricaria chamomilla* L., *Artemisia arborescens* L. etc. [31,32,33].

The aim of this study was to perform a phytochemical analysis of the Romanian *C. intybus* aerial parts extract and to assess the antioxidant, anti-inflammatory, cardioprotective and nephroprotective effects, for a scientifically based medicinal use of the indigenous plant species.

## 2. Results and Discussions

### 2.1. Phytochemical Analysis

*C intybus* extract had high polyphenols content (polyphenolic acids and flavonoids) with antioxidant activity, due to the molecular structure that is characterized by numerous hydroxyl groups [34].

The total polyphenols content (TPC), total flavonoids content (TFC) and total caffeic acid derivates content (TCAD) of *C. intybus* aerial parts methanol extract (CHME) and tincture (CHT) were determined, and the results are summarized in Table 1.

Phenolic compounds are molecules with high potential to neutralize free radicals. We suppose that the summation of TFC and TCAD is not near the TPC, because the possible presence of some reducing interferants, representing limitations of the Folin–Ciocalteu method and also because the other polyphenols present in C. intybus (coumarins, lignans, tannins, mentioned in the literature), which can contribute to the total antioxidant capacity.

Our results showed a high polyphenols content, better extracted in methanol than in ethanol 70%. The results are comparable to previous screenings on chicory aerial parts methanol extracts by Jasim et al. with a TPC of 20.4 ± 0.11 mg GAE/g d.w. [35]. According to Malik et al. similar results of TPC were obtained: 25.93 ± 0.20 mg GAE/g d.w. for methanol leaves extract and 21.01 ± 0.47 mg GAE/g for ethanol leaves extract [36]. Another study of Shad et al. reported values of 20.90 ± 0.21 mg GAE/g for methanol stems extract and 25.20 ± 0.26 GAE/g for methanol leaves extract. Jancic et al. obtained lower results in ranges of 1.05 ± 0.02 to 3.73 ± 0.04 mg GAE/g for methanol/trifluoroacetic acid extract of leaves [37,38]. Different values of TPC were reported by Dalar et al. on chicory from eastern Anatolia, using lyophilized ethanol leaves extract (70.6 ± 2.4 mg GAE/g extract) and also by Abbas et al. (85.00 ± 6.23 mg GAE/g extract) [39,40].

Previous research on the wild chicory grown in southern Romania analysed the polyphenols in different organs of the plant in methanolic extracts. Our results were higher than those reported by our colleagues (11.80 mg GAE/g d.w.) [41]. We attribute this to different sources of the vegetable material. The analyzed biological vegetable material used in our study was gathered from a mountain region (altitude 1150 m) in the center of the country characterized by lower temperatures and higher levels of precipitation. Contrary, in the previously mentioned article the vegetable material was collected from lower altitudes, a dry climate with higher temperatures and less precipitation.

The TFC determination revealed similar results to those obtained by other authors [35,40]. Shad et al. obtained lower values for TFC determined in methanol extract of stems (0.8 ± 0.03 mg CE/g d.w.) and leaves (1.00 ± 0.02 mg CE/g d.w.) [37]. Higher values of TFC were obtained by Malik et al. on methanol (13.5 ± 0.70 mg RE/g d.w.) and ethanol extracts (8.49 ± 0.08 mg RE/g d.w.) from chicory leaves [36].

To the best of our knowledge the TCAD content was evaluated for the first time and the concentration was higher in methanol extract.

### 2.2. HPLC-UV-MS (High-Performance Liquid Chromatography—Ultraviolet—Mass Spectrophotometer)Separation

In this study polyphenolic compounds were analyzed by HPLC-MS (High-performance liquid chromatography - Mass spectrophotometer) with the purpose of evaluating the qualitative and quantitative composition of the Cichorii herba methanolic extract and tincture. Two HPLC-MS methods were employed, the first for the general quantification of polyphenolic compounds and a more specific method for the quantification of cichoric acid.

The obtained results (presented in Table 2) show a composition of polyphenolic acids with the major compound cichoric acid followed by chlorogenic acid and caftaric acid. Also, flavonoid compounds were found, and the main quantified compound was isoquercitrin, followed by quercitrin and subsequently rutin. Luteolin and apigenin were quantified as well.

The assessment of cichoric acid was made considering the reported data in the literature regarding its presence in *Cichorium* species, as an important active principle with bioactive effects, such as antimicrobial, anti-inflammatory, or preventing insulin resistance in diabetes [42,43,44].

The results obtained show important quantities of cichoric acid in both samples (Table 2) as the major polyphenolic compound. The CHME was richest in cichoric acid compare to the CHT.

Similar results were obtained by HPLC/DAD/MS (High-performance liquid chromatography—Diode array detection—Mass Spectrophotometer) on ethanol extracts of wild chicory fresh leaves by Innocenti et al. [8]. Other studies reveal that cichoric acid is the most abundant compound in chicory from the Chioggia region (0.33–0.61 mg cichoric acid/g d.w.) and from the Treviso region (1.40–1.53 mg cichoric acid/g d.w.) respectively, representing between 10–15% of the total polyphenols content [8]. Comparable results were also obtained on 70% ethanol extracts of aerial parts of *C. intybus* with a range of concentrations between 0.87–6.14 mg/g dry plant [45,46]. Dalar et al. reported quantities of 16.2 mg/g cichoric acid in 80% ethanol extracts of *C. intybus* leaves [39]. Fresh chicory contained 21.30 mg/g cichoric acid, as reported by Jurgonsly et al., while another study conducted by Bahry et al. found that ethanol: acid acetic extracts of leaves contain 0.37 mg/g of cichoric acid [43,44]. Methanol extracts of aerial parts of *C. intybus* (99:1 methanol: formic acid) rank cichoric acid between 29.85–97.22 mg/100 g fresh weight, whilst another research quantified cichoric acid from methanol: formic acid (95:5) to a range of 58–186 mg/100 g fresh weight [47,48].

### 2.3. The Antioxidant Activity

The antioxidant capacity was assessed by two in vitro methods: 2,2–diphenyl-picrylhydrazil (DPPH^●^) and ferric-reducing antioxidant power (FRAP). The DPPH^●^ assay is based on the capacity of antioxidant to scavenge free radicals, whereas the FRAP assay is based upon ferric ion reducing activity. Although both assays evaluate the antioxidant activity, the comparison of results can give different outcomes based upon the choice of method [49,50]. For example, whilst the flavanols have a chemical structure fit for free radical scavenging, the antioxidant activity of anthocyanins is sensitive for the FRAP method [51].

Phenolic compounds are considered of major relevance in the total antioxidant capacity for a lot of medicinal plants. The chemical structure of the phenolic compounds, (more exactly the number of available hydroxyl groups) and their concentration are related to the antioxidant capacity of the extracts. The mixture of phenolic compounds present in extracts can determine different supplementary effects such as synergistically, additively or antagonistically actions, that influence the total capacity of the extract to neutralize free radicals. Both phenolic acids and flavonoids are known as antioxidant compounds, but their ability depends not only on the quantities but on the chemical structure also [52]. The DPPH radical scavenging activity of the extracts was modest (164.98 ± 5.93 µg/mL for the CHME and 336.35 ± 11.77 µg/mL for the CHT), but in accordance with other data [53]. Previous studies have reported even higher values (over 700 µg/mL), while several authors have obtained a better antioxidant activity for the hydroalcoholic extract of chicory leaves [54].

In our experiments, the CHME of chicory aerial parts has demonstrated a good ferric ion-reducing antioxidant capacity with a value of 896.68 ± 27.79 µM TE/g higher than CHT with a value of 594.62 ± 18.43 µM TE/g.

Antioxidant activity by FRAP method of ethanol leaves extract was evaluated by Dalar et al. as 611.00 ± 15.1 μmol Fe^+2^/g d.w. [39]. According to Malik et al. the FRAP assay results of methanol and ethanol leaves extracts of wild *C. intybus* were evaluated at 1254 ± 0.002 μmol Fe^+2^/g d.w. and 1049.73 ± 0.002 μmol Fe^+2^/g d.w. respectively [36].

Many studies revealed that antioxidant capacity is attributed to a combination of polyphenols, as was considered in our study because the total polyphenolic content of the *C. intybus* extracts was correlated with the antioxidant effects [55].

Even if the CHME analysis showed better results for polyphenols content and in vitro antioxidant activity, further in vivo pharmacological tests were conducted with CHT, for laboratory animals’ safety reasons.

### 2.4. Pharmacological Studies

#### 2.4.1. Cardioprotective Effects Evaluation

Myocardial ischemia (MI) is characterized by the imbalance between the coronary blood supply and the quantities of oxygen required for the proper function of the heart [56]. The blood flow reduction leads to tissue hypoxia, inflammation, oxidative stress, and cell death. For our experiment an isoprenaline (ISO)-induced myocardial ischemia model was used. ISO is a non-selective β1 and β2 adrenergic agonist, with a positive chronotrope and inotrope effect causing tachycardia and systolic blood pressure increase, predisposing the subject to cardiac arrhythmias. On myocardial β1 receptors it also has a pronounced positive effect. In the peripheral circulation ISO acts on arteriolar smooth muscle β2 receptors causing mild vasodilatation, with diastolic blood pressure decrease [57]. In high doses, ISO causes MI and prompt cardiac necrosis by several mechanisms: myocardial hyper function, increased intracellular Ca^++^ charge, and oxidative stress. The oxidative stress results from several metabolic products of isoproterenol, and free radicals from the injured tissue [58].

The free radicals, such as superoxide anions, hydroxyl radical, lipid peroxyl, and lipid peroxide, are unstable and highly reactive molecules, attacking molecules such as proteins, lipids and DNA [59]. Consequently, in ISO-induced MI oxidative stress may cause additional cell injury and tissue damage extension [60].

**Figure 1:**
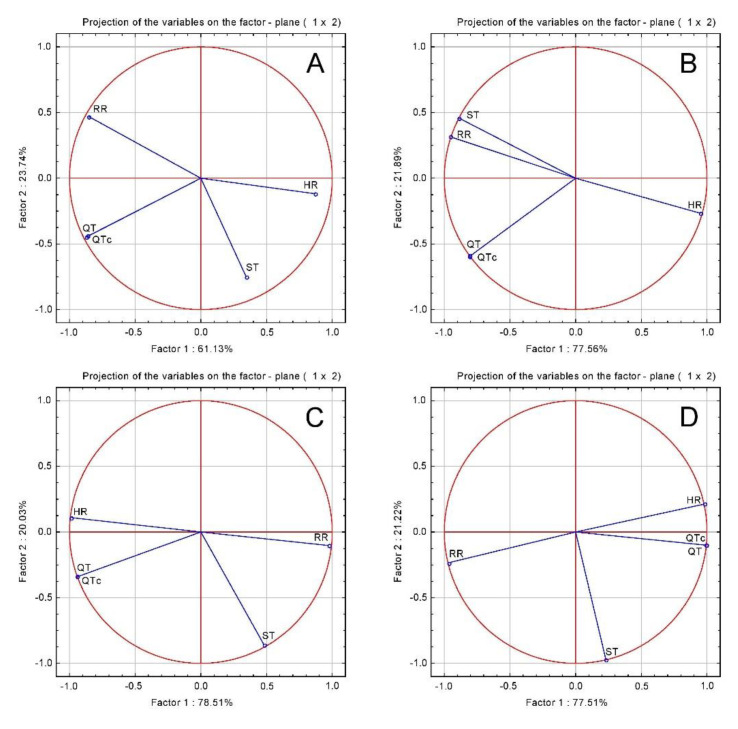
ECG parameters principal component analysis (PCA) results: (**A**) ISO group; (**B**) CHT; (C) CHT 1:1; (**D**) CHT 1:3.

The cardioprotective effects of the chicory extracts displayed by the serum cardiac marker enzymes, AST, ALT and CK-MB levels were summarized in Table 3 and by ECG (electrocardiogram) parameters presented in Table 4. The ISO-induced heart injury was demonstrated by the high serum AST, ALT and CK-MB and ECG heart rate (HR) and ST (interval of electrocardiogram) segment changes. *C. intybus* treatment lowered myocardial enzymes suggesting that it has cardioprotective effect by reducing cell injury. The treatment had no significant effect on the ECG parameters (Table 4 and Figure 1).

Oxidative stress was evaluated systemically by global tests, such as TOS, OSI and TAC, and by specific tests, such as MDA, SH and NOx (Table 5). When evaluation oxidative stress, in ISO group there was an important reduction of total antioxidant capacity (TAC) and a significant increase of total oxidative status (TOS) and oxidative stress index (OSI). CHT reduced the oxidative stress by a slight increase of TAC (*p* ˂ 0.05) and a significant decrease of TOS and OSI (*p* ˂ 0.001). The decrease of malondyaldehide (MDA) levels made a small contribution (*p* < 0.05) to oxidative stress reduction. The concentrations of total nitrites and nitrates (NOx) and total thiols (SH) were not influenced by any CHT dilutions.

Taken together, these results showed that *C. intybus* tincture has antioxidant effect in ISO-induced MI by reducing the oxidants and increasing the antioxidants. Moreover, the oxidant reduction is not associated with lipoperoxides and NO reduction. These results are important because systemic oxidative stress reduction decrease the risk of systemic complications in MI. Because myocardium is an organ that is vulnerable to oxidative damage due to the lack of antioxidant systems, systemic oxidative stress reduction may diminish the myocardial injury during MI [61].

Our phytochemical analysis of *C. intybus* tincture found an important concentration of polyphenols, especially flavonoids and cichoric acid. These phenolic compounds are largely involved in the antioxidant activity of the extracts with better results in the in vivo experiments than in vitro. Studies found in literature, comparing the in vitro and in vivo antioxidant effects of a vegetable extract, have revealed that some extracts manifest antioxidant activity both in vitro and in vivo, but for some extracts, the in vitro activity does not correlate with the in vivo antioxidant capacity. This can be related to the possible differences between the chemical structure of the phenolics, the free radicals used in the in vitro methods, and the complexity of the physiologically substances that produce oxidative effects in the organism of the animal [62].

Studies analyzing the flavonoids’ cardioprotective effects reported an inverse association between high doses of flavonoids and coronary heart incidence and mortality [63]. The role of quercitrin and of the aglycones luteolin and apigenin in heart-related diseases prevention was assessed in an extensive study [64]. Rutin reduced cardiac hypertrophy and, consequently, cardiac remodeling [65]. Also, isoquercitrin has an important role as an antioxidant in myocardial oxidative stress [66].

Cichoric acid was studied as a possible indirect cardioprotector due to its vasorelaxant activity on an isolated rat aorta against contractions induced by nor-epinephrine [67]. Also, cichoric acid was considered a cardioprotector in Tibetan yaks exposed to high altitude induced hypoxia by increasing TAC and glutathione and by decreasing MDA levels [68].

Due to the extended myocardial injury, an inflammatory response is associated with this. One of the most important factors involved in the regulation of the acute phase inflammatory response is the nuclear factor kappa B (NF-kB). By reducing NF-kB and the activation of NF-kB signaling, *C. intybus* tincture had an anti-inflammatory effect (*p* ˂ 0.01), leading to cytokines and various pro-inflammatory genes expression inhibition [69,70].

All of these results indicated that *C. intybus* tincture has cardioprotective effects due to the antioxidant and anti-inflammatory activity. These effects were correlated with the the improvement of the myocardial enzymes.

**Figure 2:**
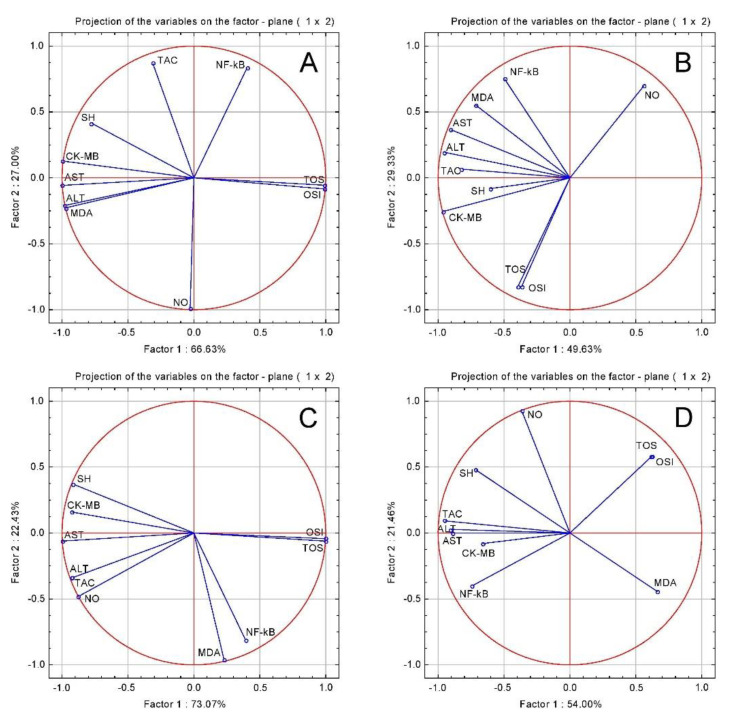
Oxidative stress tests and cardiac function tests PCA results: (**A**) ISO group; (**B**) CHT; (**C**) CHT 1:1; (**D**) CHT 1:3.

In order to evaluate the relationship between the parameters, a principal component analysis (PCA) correlation circle was employed (Figure 2). In the CONTROL, CHT, CHT 1:1 and CHT 1:3 groups there was a significant positive correlation between the transcription factor NF-Kb, oxidative stress markers and cardiac enzymes markers, indicating that myocardial ischemic injury was associated with inflammation and oxidative stress.

#### 2.4.2. Nephroprotective Effects Evaluation

The *C. intybus* tincture effect on acute kidney injury (AKI) induced by gentamicin (GENTA) was also evaluated in the present study. The renal function test serum creatinine (SCr), serum urea (SU), urine creatinine (UCr), urine urea (UU) and creatinine clearance (CrCl) are summarized in Table 6. The serum oxidative stress markers and NF-Kb levels are outlined in Table 7.

In the GENTA group, animals renal function parameters were significantly increased due to the gentamicine nephrotoxicity [71]. SCr, UCr, SU and UU were lowered by CHT pre-treatments in a concentration-dependent way, the higher concentration (CHT) having the better nephroprotection activity. CrCl did not change significantly due to the CHT pre-treatment (*p* > 0.05).

CHT 1:1 and CHT 1:3 reduced oxidative stress by lowering TOS (*p* ˂ 0.001) and OSI (*p* ˂ 0.001), and CHT by reducing TOS (*p* ˂ 0.05), OSI (*p* ˂ 0.05), and NOx (*p* ˂ 0.05). The CHT samples had no important effect on MDA and SH (*p* > 0.05). CHT lowered the transcription factor NF-Kb in a reversed concentration-dependent way, the lower concentrations being more efficient inhibitors.

**Figure 3:**
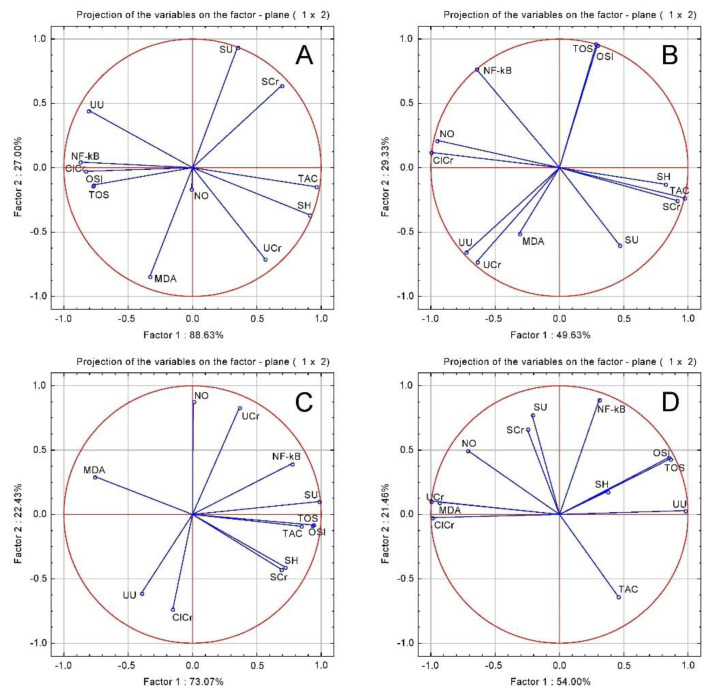
Oxidative stress and renal function tests PCA results: (**A**) GENTA group; (**B**) CHT; (**C**) CHT 1:1; (**D**) CHT 1:3.

In order to evaluate the relationship between the parameters a PCA was performed (Figure 3). In the GENTA group, SCr, SU, UCr and UU correlated positively with TOS, OSI, NOx, MDA, and Nf-kB, and negatively with SH and TAC. In the CHT groups, the renal function tests were negatively correlated with TOS, OSI, NOx and Nf-kB.

Despite the fact that the gentamicin renal toxicity is documented, the mechanisms are not completely understood. A recent hypothesis suggests a mechanism based on oxidative stress and inflammation [72]. In the present study, renal function, oxidative stress and inflammation were evaluated in serum and in urine. In rat gentamicin-induced AKI, the parameters SC, UC, SU, UU, CrCl were consistent with AKI [71]. Increased TOS, OSI, NO and MDA, plus TAC and SH levels reduction proved the involvement of the oxidative stress. Moreover, the high Nf-Kb indicated the existence of an inflammatory response.

Serum and urine creatinine and urea were lowered by CHT pre-treatments in a reversed concentration-dependent way, the lower concentration having the better nephroprotective activity. CrCl did not change significantly due to the CHT pre-treatment.

In AKI animals, oxidative stress was reduced by the *C. intybus* tincture by lowering TOS, OSI, and NOx, plus by increasing TAC, in a reversed concentration-dependent way, CHT 1:1 having the lowest antioxidant activity. The *C. intybus* tincture did not influence lipids peroxidation and thiols because the samples had no important effect on MDA and SH.

*C. intybus* tincture had also anti-inflammatory effects by lowering the transcription factor NF-Kb in a reversed concentration-dependent way, the lower concentrations being a more efficient inhibitor.

*C. intybus* contains caffeic acids and flavonoids. Many studies regarding flavonoids (as rutin, quercetin, luteolin and apigenin) show diuretic and nephroprotective properties [73,74,75,76]. Also, polyphenolic acids have been studied for major role in reestablishing renal function subsequently to oxidative stress [77]. Cichoric acid renal effects were evaluated in a previous study, showing that it can prevent methotrexate-induced AKI by decreasing ROS (reactive oxygen species)-induced activation of NF-κB/NLRP3 inflammatory pathway [68].

These observations were in agreement with our results, as *C. intybus* contains compounds that contribute to pharmacological activities. Within *C. intybus* composition, cichoric acid stands out, being one of the polyphenol acids with the highest concentration, especially in aerial parts [8].

From our experiment we concluded that the diluted tincture, CHT 1:1 had higher nephroprotective activity. This observation can be related to the fact that some plant extracts with high polyphenol content can result in an oxidant and pro-inflammatory effect, and just lower concentrations have antioxidant and anti-inflammatory activity in vivo.

In this study we have pointed out that *C. intybus* tincture has cardioprotective effects on isoprenaline-induced MI and nephroprotective effects on gentamicine-induced AKI, by reducing the systemic oxidative stress and the inflammatory response.

Studies analyzing the antioxidant activity of plant extracts using in vitro and in vivo tests show that these are not always correlated [78]. In our case, the in vitro and in vivo antioxidant activities were not correlated, the antioxidant activity in vitro was modest whilst the in vivo studies showed a good antioxidant capacity.

## 3. Materials and Methods

### 3.1. Chemicals and Reagents

The following standards used in the phytochemical analysis of LC-MS were purchased from Sigma-Aldrich (Schnelldorf, Germany): caftaric acid (>97%), chlorogenic acid (>95%), cichoric acid (>95%), isoquercitrin (quercetin 3-d-glucoside) (≥98%), quercitrin (quercetin 3-rhamnoside) (≥78%), luteolin (≥98%), apigenin, syringic acid (≥95%), protocathehuic acid (3,4-dihydroxybenzoic acid) (≥97%), vanillic acid (≥97%), hispidulin(≥95%). Methanol, ammonium acetate, acetonitrile, petroleum ether, chloroform, hydrochloric acid, acetic acid, potassium hydroxide, Folin-Ciocâlteu reagent, were purchased from Merck (Darmstadt, Germany). Sodium carbonate, sodium acetate trihydrate and anhydrous aluminium chloride were purchased from Sigma-Aldrich (Schnelldorf, Germany). TPTZ agent, DPPHagent and trolox were acquired from Sigma-Aldrich (Schnelldorf, Germany); gallic acid and rutin from (Fluka Chemie GmbH, Buchs, Switzerland). Methanol p.a., ethanol 96%and dichloromethane were purchased from Chemical Company (Iași, Romania) and iron chloride fromMerck (Darmstadt, Germany). The commercial biochemistry kits for the pharmacological investigations (kit CK-MB-LQ.Anti CK-M.Immunoinh.; kit GOT/AST-LQ. IFCC. Enzymatic–UV; GPT/ALT-LQ. IFCC. Enzymatic–UV; kit UREA-LQ. Urease-GLDH. Kinetic; kit CREATININE-J. J) were purchased from S.C. DG DIAGNOSTICS S.R.L Cluj-Napoca. The isoprenaline used to induce myocardial ischemia was purchased from Sigma-Aldrichand the gentamicin 80 mg/2 mL (KRKA, Novo mesto, Slovenia) used for renal failure was purchased from a local pharmacy.

### 3.2. Plant Material and Extraction Procedure

The vegetable material, *C. intybus* aerial parts (*Cichorii herba*), was collected from Alba County, Lat. 45.643412/Long. 23.640752, Romania, during the flowering stage, in August 2018 from spontaneous flora. A sample of the vegetable material is held in the herbarium of the Pharmacognosy Department (voucher number 114). The plant material was air-dried previous extraction. The results of chemical analysis were expressed per g dry plant material (d.w.).

The methanol extracts were obtained from 5 g plant powder (aerial parts previously extracted with dichloromethane in Soxhlet) and 50 mL methanol 70%, for 30 min at 60 °C (CHME). The tincture (1:10) was obtained from 50 g of herbal material and 500 mL 70% ethanol by maceration at room temperature for 7 days (CHT) [79].

The CHME and CHT were used to determine total polyphenolic content, total flavonoid content, total caffeic acid derivates content, for HPLC determinations and antioxidant assays (DPPH^•^ and FRAP methods).

Due to safety issues concerning methanol toxicity, the tincture was selected for the pharmacological experiments.

For the assessment of the cardioprotective and nephroprotective activities, *C. intybus* tincture (corresponding to 1 mg dry weight plant material/10 mL) was used, as well as two dilutions of the tincture, obtained with distilled water, CHT 1:1 (0.5 mg dry weight plant material/10 mL) and CHT 1:3 (0.25 mg dry weight plant material/10 mL).

### 3.3. Total Phenolic Content Determination

The TPC of *C. intybus* extracts was spectrophotometrically determined by Folin-Ciocâlteu method with some modifications [80]. Briefly, 2 mL of both methanol and ethanol extracts were diluted in a 25 mL calibrated flask with the same solvent. 2 mL of each solution were mixed with 1 mL Folin-Ciocâlteu reagent, 10 mL of distilled water and diluted to 25 mL with a solution of sodium carbonate (290 g/L). The samples were stored in darkness for 30 min., and then the absorbance was measured at 760 nm using a Cary 60 ultraviolet–visible (UV–Vis) spectrophotometer from Agilent Technologies (R^2^ = 0.999). TPC was expressed as gallic acid equivalents (GAE)/g dry plant material [81]. All the TPC determinations were realized in triplicate.

### 3.4. Total Flavonoid Content Determination

The TFC of *C. intybus* extracts was determined spectrophotometrically using AlCl_3_ as a color reagent. In summary, 10 mL of each extract were diluted to 25 mL using methanol, 5 mL were mixed with sodium acetate (5.0 mL, 100 g/L), aluminium chloride (3.0 mL, 25 g/L), and further diluted with methanol up to 25 mL in a calibrated flask [82]. The absorbance was measured at 430 nm and the results were expressed as rutin equivalents (RE)/g dry plant material (R^2^ = 0.999). All experiments were realized in triplicate.

### 3.5. Total Caffeic Acid Derivates Content Determination

The caffeic acid derivates content (CADC) of CHME and CHT was determined spectrophotometrically using Arnow reagent. Briefly, 10 mL of both extracts were diluted with methanol at 25 mL in a volumetric flask, from which 5 mL were taken and diluted with ethanol 50% at 10 mL. 1mL of this solution was mixed with 1 mL of HCl 0.5 N, 1 mL of Arnow reagent and 1 mL NaOH 1 N and further diluted to 10 mL [83]. The absorbance was measured at 500 nm and the results were expressed as cichoric acid equivalents (CAE)/g dry plant material (R^2^ = 0.994). All determinations were realized in triplicate.

### 3.6. Evaluation of In Vitro Antioxidant Capacity

#### 3.6.1. DPPH Radical Scavenging Activity

DPPH radical scavenging array is a spectrophotometric quantitative method based upon the reaction between the antioxidant compounds in the extracts samples and DPPH^●^ reagent in an alcoholic solution 2 mL (at different concentrations) of both CHME and CHT extracts were added to 2 mL of a 0.1 gL^−1^ DPPH^●^ methanol solution. After 30 min in a thermostatic bath at 40 °C, the variation of the absorbance was measured at 517 nm (R^2^ = 0.997). The percent of DPPH^●^ scavenging ability was calculated as follows: DPPH scavenging ability % = (A control − A sample/A control) × 100, where A control is the absorbance of DPPH^●^ radical + methanol (containing all reagents except the sample) and A sample is the absorbance of DPPH radical + sample extract. The percentage of DPPH decrease was quantified in Trolox equivalents (TE). The DPPH radical scavenging activity of C. intybus extracts was expressed as IC_50_ (µg/mL). IC_50_(half maximal inhibitory concentration) value relates to the antioxidant capacity; if IC_50_ ≤ 50 µg/mL, the samples have good antioxidant capacity, if IC_50_ is contained within the range of (50–100) µg/mL, the antioxidant capacity of the extracts is moderate and if IC_50_ ≥ 200 µg/mL the samples have low to negligible antioxidant capacity [84,85,86]. The experiments were realized in triplicate.

#### 3.6.2. Ferric-Reducing Antioxidant Power Assay

The FRAP method is a spectrophotometric method that evaluates the antioxidant capacity of the samples based on the reduction of the ferric complex2,4,6-tri(2-pyridyl)-1,3,5-triazine(Fe(II)-TPTZ) to ferrous complex (Fe(III)-TPTZ) determining a variation of the color that can be measured [87]. The FRAP reagent consists of a mixture between 2.5 mL of 10 mM TPTZ solution in 40 mM HCl mixed with 2.5 mL 20 mM ferric chloride solution and 25 mL of acetate buffer at a pH of 3.6. A volume of 4 mL of each ME and EE extract were diluted with water to 1.8 mL and mixed with 6 mL of FRAP reagent (R^2^ = 0.992). Trolox was used as reference. The absorbance was measured at 450 nm [88].

### 3.7. HPLC-UV-MS Separation

The analysis was conducted using an Agilent Technologies 1100 HPLC Series system (Agilent, Santa Clara, CA, USA) equipped with G1322A degasser, G13311A binary gradient pump, column thermostat, G1313A auto sampler and G1316A UV detector. The HPLC system was coupled to an Agilent 1100 mass spectrometer (LC/MSD Ion Trap SL). A reverse-phase analytical column (Zorbax SB-C18 100 × 3.0 mm i.d., 3.5 μm particles) was used for the separation at the work temperature of 48 °C. The detection of the compounds was performed in both UV and MS mode The MS system functioned using an electro spray ion source in negative mode. ChemStation and Data Analysis software from Agilent were used for the chromatographic data processing. The flow rate was 1 mL/min and the injection volume was 5 µL. The MS traces/spectra of the analyzed samples obtained in the experiment were compared to spectra from library, which allowed positive identification of compounds. The UV trace was used for quantification of identified compounds from MS detection. The limit of quantification for the compounds was 0.5 μg/mL, and the limit of detection was 0.1 μg/mL. The detection limits were calculated as minimal concentration producing a reproductive peak with a signal-to-noise ratio greater than three. Quantitative determinations were performed using an external standard method. Calibration curves in the 0.5–50 μg/mL range with good linearity (R^2^ > 0.999) for a five point plot were used to determine the concentration of polyphenols in plant samples [84,89]. Analysis of cichoric acid from *C. intybus* extracts was performed using a newly developed procedure of liquid chromatography coupled with mass spectrometry detection. This method is characterized by rapid analysis (<1 min per sample), and high specificity due to mass spectrometry detection. The stock solution of cichoric acid (1 mg/mL) was prepared by dissolving the reference standard in methanol and was used to prepare calibration standards with concentrations of 0.75, 1.5, 3.0, 7.5, and 15 μg/mL (three control standards 0.75, 3.0, and 15 μg/mL were used to assess the precision and accuracy of the method). Bidistilled water was used in order to prepare the calibration standards. The mobile phase consisted in 95/5 (*v*/*v*) ammonium acetate, 1 mM in water and acetonitrile, isocratic elution, and mobile phase flow rate of 1 mL/min. The mass spectrometer operated in negative mode and nitrogen was used as a nebulizing and dry gas. The nebulizer was positioned at 65 psi with the dry gas flow at 12 L/min at 350 °C. The mass spectrometer recorded the specific transition of cichoric acid *m/z* 473 > *m/z* 293 + *m/z* 311. Cichoric acid had a time of retention of 0.51 min. The concentration of cichoric acid was determined automatically using peak area and the external standard method by the instrument data system (Quant Analysis 1.7 software, Brucker Daltonics, Darmstadt, Germany). Linear regression was used in order to obtain the calibration curve using a 1/Y2 weighting scheme. The lower limit of quantification was established at 0.75 μg/mL. At quantification limit, the method precision (expressed as coefficient of variation (CV) %) was 6.8%, and accuracy (expressed as relative difference between obtained and theoretical concentration, bias %) was 5.3%, respectively (R^2^ > 0.999) [90].

### 3.8. Pharmacological Evaluation

#### 3.8.1. Experimental Animals

The experiments were performed on adult male Winstar albino rats, weighing 200–250 g. The animals were bred in the “Iuliu Hațieganu” University of Medicine and Pharmacy Animal Facility, and were kept under controlled conditions (12 h night/day cycle, temperatures of 21–22 °C and humidity of 50–55%) with free access to standard pellets based diet (Cantacuzino Institute, Bucharest, Romania) and water ad libitum. All the animals were sacrificed by cervical dislocation at study completion under general anesthesia. The experimental design was approved by the Institutional Animal Ethical Committee (IAEC) of the “Iuliu Hațieganu” University of Medicine and Pharmacy Cluj-Napoca and by the National Sanitary Veterinary and Food Safety Agency (nr. 170/13.07.2019).

#### 3.8.2. Protocols

##### Cardioprotective Effects Evaluation Protocol

The animals were divided into 5 groups (*n* = 5): negative control (CONTROL), isoprenaline (ISO) and *C. intybus* tincture (CHT) (100 mg/1mL *w*/*v*) administrated in three dilutions (CHT 100%, CHT: solvent 1:1 = 50%, CHT: solvent 1:3 = 25%). For seven days the animals received by gavage (orally—p.o. 1 mL/d) water in groups CONTROL and ISO, respectively the three dilutions of extract in the CHT groups. Excepting the CONTROL group, on days 8 and 9 animals received isoprenaline (subcutaneously—s.c. 150 mg/kg body weight—b.w.) in order to induce experimental MI [55]. In day 10 ECG was recorded and blood samples were collected by retro-orbital puncture under general anesthesia induced by a mixture of ketamine (70 mg/kg b.w.) and xilazine (10 mg/kg b.w.) [91]. Serum was separated and stored at −80 °C until the oxidative stress and cardiac markers analysis.

ECG was recorder in lead DII (right forelimb to left hind limb) with a Biopac MP150 system. The apparatus was calibrated at 1 m V/1 cm with a speed of 50 mm/s. Heart rate (beats/min), RR intervals (msec), QT intervals (msec), and ST segment changes (mV) were measured. Corrected QT interval (QTc) according to Bazett formula was also calculated [55].

Cardiac markers, aspartate transaminase (AST), alanine aminotransferase (ALT) and the creatinine kinase myocardial band (CK-MB), were assessed using commercial kits.

##### Nephroprotective Effects Evaluation Protocol

The animals were divided into 5 groups (n = 5): negative control (CONTROL), gentamicin group (GENTA), and *C. intybus* tincture (CHT) (100 mg/1 mL *w*/*v*) administrated in three dilutions (CHT =100%, CHT 1:1 = 50%, CHT 1:3 = 25%). For seven days the animals received water by gavage in groups CONTROL and GENTA, respectively, and the three dilutions of extract in the CHT groups [92]. Excepting the CONTROL group, on days 7 and 8 animals received gentamicin (s.c. 400 mg/kg b.w./d) for nephrotoxic acute kidney failure induction [93]. The 24 h urine was collected between days 8 and 9. On day 9 blood samples were also collected by retro-orbital puncture under general anesthesia (ketamine 70 mg/kg b.w.) and xilazine 10 mg/kg b.w.) [91]. Serum was separated and stored at −80 °C until oxidative stress and renal function evaluation.

##### Oxidative Stress Parameters Evaluation

TOS was assessed using a colorimetric method based on the oxidation of a ferrous ion to a ferric ion in the presence of various oxidant species [94]. The results were expressed in µmol H_2_O_2_ equiv/L. TAC was measured using a colorimetric assay described by Erel and expressed as mmol Trolox equiv./L. [95]. OSI was calculated representing the ratio between TOS and TAC [96]. As a lipid peroxidation marker, MDA was determined using the thiobarbituric acid assay. The MDA serum concentration was expressed as nmol/mL [97]. The serum NO concentration was assessed using the Griess reaction and expressed as nitrite µmol/L (NOx) [98]. Serum total thiols were expressed as mmol GSH/mL and were determined using Ellman’s reagent [99].

Serum and urine creatinine were determined according to the manufacturer instructions (AMEDA Labordiagnostik GmbH, Graz, Austria), and CrCL was calculated according to the formula: CrCL = (urine creatinine diuresis)/plasma creatinine.

##### ELISA (Enzyme-Linked Immunosorbent Assay) 

The NF-Kb was determined using a NF-kB (Nuclear Factor Kappa B) ELISA KIT, (ER1186, Fine Biotech, and Wuhan, China) according to the manufacturer instructions.

#### 3.8.3. Statistical Analyzes

The results were expressed as means and standard deviation. The data were compared by using a one-way analysis of variance (ANOVA) test and post-hoc Bonferroni–Holm test. The correlation between the parameters of the same group was assessed by Pearson’s coefficient (r) according to the Colton scale. The level of significance was established at *p* ˂ 0.05. Multivariate analysis of the parameters was performed using PCA. The statistical analysis was performed using STATISTICA 12.0 software.

## 4. Conclusions

The Romanian chicory, *C. intybus,* was the subject of this research, offering data regarding the chemical composition and biological properties. Among the compounds identified by HPLC-UV-MS assay, the major polyphenol was cichoric acid, in both methanol extract and tincture The antioxidant capacity was evaluated both in vitro and in vivo with different outcomes related to polyphenols total content suggesting complex relationships between the compounds. The cardioprotective and nephroprotective properties were investigated through antioxidant and anti-inflammatory mechanisms on rats, revealing promising results in treating acute myocardial ischemia and acute renal failure in rats. Our findings give new directions for further studies in order to extend the scientific basis for the therapeutic uses of an indigenous species.

## Figures and Tables

**Table 1:** The polyphenol content of *C. intybus* extract.

Sample	TPC (mg GAE/g d.w.)	TFC (mg RE/g d.w.)	TCAD (mg cichoric acid/g d.w.)
CHME	23.94 ± 0.42 *	5.06 ± 0.06 *	8.90 ± 0.15 *
CHT	14.34 ± 0.28 *	3.68 ± 0.04 *	2.90 ± 0.04 *

Note: values are expressed as mean of 3 determinations ± SD. * *p* < 0.01.

**Table 2:** Phenolic compounds identified in *C. intybus* extracts by HPLC-UV-MS.

Polyphenolic Compounds	[M-H]- *m/z*	RT ± SD (min)	CHME Concentration µg/g d. w.	CHT Concentration µg/g d. w.
Caftaric acid	311	3.52 ± 0.05	742.81 ± 28.73	-
Chlorogenic acid	353	5.62 ± 0.05	910.69 ± 36.15	-
Cichoric acid	473	15.96 ± 0.13	18450.00 ± 732	6122.58 ± 232.10
Isoquercitrin	463	19.60 ± 0.10	427.30 ± 16.43	296.31 ± 11.85
Rutin	609	20.20 ± 0.15	14.51 ± 0.66	15.99 ± 0.56
Quercitrin	447	23.64 ± 0.13	104.61 ± 4.01	54.13 ± 1.95
Luteolin	285	29.10 ± 0.19	3.98 ± 0.12	21.96 ± 0.82
Apigenin	269	33.10 ± 0.15	-	6.77 ± 0.23

Values are the mean ± SD (*n* = 3).

**Table 3:** Serum cardiac injury markers in rat isoprenaline-induced myocardial ischemia.

Groups	AST (UI/L)	ALT (UI/L)	CK-MB (UI/L)
CONTROL	35.32 ± 10.94	29.10 ± 9.20	7.26 ± 2.28
ISO	49.94 ± 16.71	40.04 ± 14.58	12.10 ± 2.15
CHT	32.71 * ± 8.60	28.14 * ± 9.07	11.55 ± 2.29
CHT 1:1	38.43 * ± 5.33	33.25 * ± 7.53	9.35 ± 2.45
CHT 1:3	37.58 * ± 10.25	32.99 * ± 12.70	8.03 * ± 1.68

Note: Values are expressed as mean ± SD (*n* = 5). * *p* ˂ 0.05, versus ISO group.

**Table 4:** ECG parameters (RR, QT, QT corrected, ST intervals and heart rate) in rat isoprenaline-induced myocardial ischemia.

Groups	HR (beats/min)	RR (s)	QT (s)	QTc (s)	ST (mV)
CONTROL	215.00 ± 42.50	0.23 ± 0.02	0.08 ± 0.02	0.2 ± 0.03	0
ISO	283.50 ^##^ ± 47.95	0.21 ± 0.03	0.09 ± 0.03	0.29 ± 0.05	0.08 ^#^ ± 0.07
CHT	257.50 ± 40.10	0.24 ± 0.04	0.08 ± 0.02	0.28 ± 0.04	0.03 ± 0.00
CHT 1:1	337.75 ± 9.5	0.18 ± 0.00	0.07 ± 0.00	0.26 ± 0.02	0.04 ± 0.02
CHT 1:3	276.20 ± 48.73	0.22 ± 0.04	0.07 ± 0.01	0.26 ± 0.02	0.04 ± 0.02

Note: Values are expressed as mean ± SD (*n* = 5). ^#^ *p* ˂ 0.05, ^##^ *p* ˂ 0.01, versus CONTROL.

**Table 5:** Serum oxidative stress markers in rat isoprenaline-induced myocardial ischemia.

Groups	TOS (µM H_2_O_2_ equiv/L)	TAC (MTrolox equiv/L)	OSI	NOx (µM/L)	MDA (nM/L)	SH (mM/L)	NF-kB (ng/mL)
CONTROL	5.13 ± 0.84	1.09 ± 0.001	4.70 ± 0.77	32.67 ± 2.38	1.91 ± 0.19	0.52 ± 0.05	2.2 ± 0.22
ISO	7.43 ^##^ ± 0.22	1.09 ^#^ ± 0.000	6.83 ^###^ ± 0.20	45.51 ^###^ ± 0.73	3.41 ^##^ ± 0.48	0.39 ^#^ ± 0.03	3.42 ^#^ ± 1.18
CHT	4.92 *** ± 0.28	1.09 * ± 0.002	4.51 *** ± 0.26	44.22 ± 1.86	2.63 * ± 0.30	0.40 ± 0.04	1.72 ** ± 0.28
CHT 1:1	7.81 ± 0.23	1.09 ± 0.002	7.19 ± 0.23	44.33 ± 5.42	3.28 ± 1.02	0.40 ± 0.06	2.65 * ± 0.56
CHT 1:3	6.09 ± 1.32	1.09 ± 0.002	5.60 ± 0.22	46.57 ± 3.79	3.39 ± 0.31	0.41 ± 0.03	2.54 * ± 0.15

Note: Values are expressed as mean ± SD (*n* = 5) ^#^ *p* ˂ 0.05, ^##^ *p* ˂ 0.01, ^###^ *p* ˂ 0.001 versus CONTROL; * *p* ˂ 0.05, ** *p* ˂ 0.01, *** *p* ˂ 0.001 versus ISO group.

**Table 6:** Renal function parameters in rat gentamicin-induced acute kidney injury.

Groups	SCr mg/dL	UCr mg/dL	SU mg/dL	UU mg/dL	CrCl mL/min
CONTROL	0.72 ± 0.03	32.10 ± 1.66	23.23 ± 4.17	173.33 ± 61.28	0.60 ± 0.07
GENTA	1.24 ^##^ ± 0.71	47.26 ^#^ ± 0.28	79.04 ^###^ ± 6.55	431.83 ^###^ ± 2.12	0.27 ^###^ ± 0.02
CHT	1.03 * ± 0.11	45.15 ± 2.17	58.07 ** ± 7.29	260.00 ** ± 86.66	0.29 ± 0.06
CHT 1:1	1.08 * ± 0.11	30.77 * ± 3.89	60.32 ** ± 8.87	231.11 ** ± 100.07	0.28 ± 0.06
CHT 1:3	1.08 * ± 0.05	37.96 * ± 9.95	61.36 ** ± 6.08	317.78 * ± 50.03	0.24 ± 0.06

Values are expressed as mean ± SD (*n* = 5). ^#^ *p* ˂ 0.05, ^##^ *p* ˂ 0.01, ^###^ *p* ˂ 0.001 versus CONTROL; * *p* ˂ 0.05, ** *p* ˂ 0.01, versus GENTA group.

**Table 7:** Serum oxidative stress markers in rat gentamicin-induced acute kidney injury.

Groups	TOS (µM H_2_O_2_ equiv/L)	OSI	TAC (mmolTroloxequiv/L)	NOx (µM/L)	MDA (nM/L)	SH (mM/L)	NF-Kb (ng/mL)
CONTROL	5.13 ± 0.84	4.70 ± 0.77	1.09 ± 0.002	32.67 ± 2.38	1.91 ± 0.19	0.52 ± 0.05	2.2 ± 0.22
GENTA	7.55 ^###^ ± 1.44	6.94 ^###^ ± 1.33	1.09 ± 0.002	51.73 ^##^ ± 4.25	2.87 ^#^ ± 0.26	0.48 ± 0.02	5.81 ^###^ ± 0.10
CHT	6.06 * ± 1.89	5.57 * ± 1.73	1.09 ± 0.002	40.54 * ± 4.43	2.42 ± 0.37	0.45 ± 0.07	5.03 ± 4.94
CHT 1:1	4.01 *** ± 0.56	3.67 *** ± 0.54	1.09 ± 0.002	45.10 ± 5.34	2.83 ± 0.53	0.49 ± 0.12	2.27 *** ± 0.34
CHT 1:3	4.66 ** ± 0.73	4.28 ** ± 0.67	1.09 ± 0.002	47.28 ± 5.12	2.89 ± 0.50	0.45 ± 0.07	3.27 *** ± 1.90

Values are expressed as mean ± SD (*n* = 5). ^#^ *p* ˂ 0.05, ^##^ *p* ˂ 0.01, ^###^ *p* ˂ 0.001 versus CONTROL; * *p* ˂ 0.05, ** *p* ˂ 0.01, *** *p* ˂ 0.001 versus GENTA group.

## Data Availability

Publication ethics statement was consulted and all ethical guidances for authors were followed.
